# Perforin Expression in Peripheral Blood Lymphatic Cells of Patients Subjected to Laparoscopic or Open Cholecystectomy

**DOI:** 10.1155/2009/125152

**Published:** 2009-05-07

**Authors:** Harry Grbas, Ines Mrakovčić-Šutić, Arsen Depolo, Biserka Radošević-Stašić

**Affiliations:** ^1^Department of Surgery, Clinical Hospital Center, Medical Faculty, University of Rijeka, 51000 Rijeka, Croatia; ^2^Department of Physiology and Immunology, Medical Faculty, University of Rijeka, 51000 Rijeka, Croatia

## Abstract

Perforin-(P-) related characteristics of cytotoxic T lymphocytes and natural killer cells were investigated in peripheral blood of patients subjected to open (OC; *n* = 23) or laparoscopic cholecystectomy (LC; *n* = 21) and healthy controls (*n* = 20). Blood samples were obtained preoperatively and 24 hours after the surgeries, and the data were correlated with the intensity of cholestasis and concomitant inflammation, determined by functional hepatic tests. Postoperative differences were found to be minimal: OC decreased only the percentage of CD56^+^ cells, while LC decreased the fraction of CD8^+^P^+^ cells and augmented the mean fluorescence intensity of P in CD56 cells. Patients elected for OC had, however, higher preoperative numbers of total P^+^, CD3^+^P^+^, and CD4^+^P^+^ cells than patients elected for LC and healthy controls, while both groups of patients, preoperatively, had lower fraction of CD16^+^P^+^ and CD56^+^P^+^ cells. These changes were in high correlation with blood concentrations of CRP, AP, and ALT, emphasizing the link between the preoperative cholestasis and inflammation and P-dependent cytotoxic mechanisms.

## 1. Introduction

Since its introduction in the late 1980s, laparoscopic cholecystectomy (LC) has been used as a “gold standard” for management of symptomatic gallstone disease, replacing almost completely the open cholecystectomy (OC) [1–3]. It is commonly followed by less pain, shorter hospitalization, and faster recovery owing to the reduced magnitude of the initial surgical insult and lower systemic immune impairment [4]. However, positive CO2 pneumoperitoneum, abdominal wall distention, and peritoneal acidosis may activate the additional factors that affect the outcome of operations, leading to both the potentiation of surgical trauma [4, 5], and the beneficial immune-modulating effect, linked with the augmentation of IL-10, attenuation of TNF*α* production [6–8], and diminishing effect on surgery-induced imbalance in pro-/anti-inflammatory cytokines [9, 10]. Cellular mechanisms underlying these effects are, however, very complex, since surgical manipulation or pneumoperitoneum and laparotomy may alter the expression of 116 and 398 splenic T-cell genes, respectively, that might be involved in signal transduction, chemotaxis, and cell cycle of lymphocytes [11]. 

In the present study, we tried to analyze the perioperative changes in cytotoxic T lymphocytes (CTLs) and natural killer (NK) cells after LC and OC, since, particularly, these cells are involved in immune surveillance and have the crucial roles in the prevention or attenuation of disease through their ability to eliminate infected or transformed cells [12–15]. Their cytoplasm is rich in granules composed of complexes of proteoglycan in association with the pore-forming protein perforin (P) and a family of serine proteinases, known as granzymes that are released into the intercellular space on interaction of NK cells or CTL with a target cell. Whether granzyme entry into the target cell cytoplasm occurs directly after perforin pore formation in the plasma membrane or after endosomolysis is still debated [12–15]. According to the original model, P acts by forming a cell membrane pore through which granzymes pass into the target cell, where they cleave specific substrates that initiate DNA fragmentation and caspase-dependent or caspase-independent apoptotic cell death. However, more recently, it was proposed that granzymes might be endocytosed independently of P and that P acts to release granzymes that are sequestered in endosomes into the cytosol of the target cell. In addition, there is a possibility that P triggers Ca^2+ ^ influx, which may activate rapid coendocytosis of granzymes and P into giant endosomes, followed by P-mediated release of granzymes to the cytosol [12–15]. 

In circulating nonactivated lymphocytes, cytotoxicity is restricted to NK cells, *γ*/*δ* TCR^+^ T cells, and a subpopulation of CD8^+^ cells, which constitutively express perforin but its expression can be induced in many CD4^+^, MHC class II restricted CTL, in CD8^+^ CTL as well as in CD4^low^CD25^high^FoxP3^+^ T regulatory cells [12–15]. Moreover, the expression of P in T cells might be a useful marker for functionally activated cytotoxic cells in situ [12].

Trying to determine the early postoperative effects of classic and laparoscopic cholecystectomy on these events, in this study we analyzed the P content in the PBMNLC before and 24 hours after the operations. Furthermore, in an attempt to link these events with the intensity of preoperative cholestasis and inflammation induced by gallstones, we correlated these data with the blood concentrations of acute phase proteins and markers of cholestasis, such as bilirubin (BIL), alkaline phosphatase (AP), and hepatic enzymes. The data showed that perforin-dependent cytotoxic mechanisms are only moderately affected by different types of operations, but that the preoperative cholestasis and hepatic injury may significantly increase the number of peripheral blood cells containing the perforin.

## 2. Patients and Methods

### 2.1. Patients

In a prospective, nonrandomized study that was approved by Ethical Committee of Medical faculty, University of Rijeka, we analyzed the immune functions of 23 patients submitted to conventional, open cholecystectomy (OC) and 21 patients undergoing laparoscopic cholecystectomy (LC) for symptomatic cholecystolithiasis. Mean age for the conventional group was 61.8 years (range 31 to 85) for 13 women and 10 men. For LC, mean age was 55.5 years (range 28 to 82); this group consisted of 13 women and 8 men. Laparoscopic cholecystectomy was carried out in a standardized technique using 4 troacars and a pneumoperitoneum of 14 mm Hg. Open cholecystectomy was performed through a right subcostal incision. General anesthesia was the same in both groups. Mean operative duration was 90 minutes (range 74–139) for OC and 120 minutes (range 90–150), for LC without statistically significant difference, showing that the two groups were comparable for the demographic and surgery-related parameters (Table 1). 

**Figure 1:**
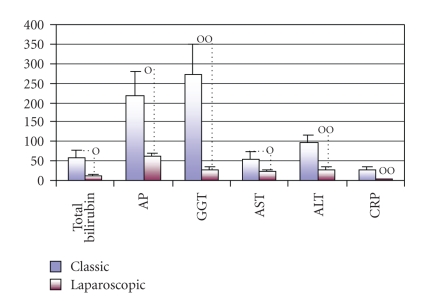
Preoperative values of total bilirubin, enzyme tests (alkaline phosphatase-AP, gamma-glutamyl transferase-GGT, aspartat aminotransferase-AST, alanine aminotransferase-ALT and C-reactive protein-CRP in sera of patients chosen for classic or laparoscopic cholecystectomy. o *P * < * .*05, oo *P * < * .*01.

There were also no statistically significant differences in the standard hematological parameters, such as red and white cells counts, hematocrit, and platelets (not shown) but group of patients chosen for classic cholecystectomy had significantly greater preoperative cholestasis (Figure 1), since laparoscopic cholecystectomy with insufflations of CO2 was avoided in patients with chronic cholecystolithiasis. Therefore, this group of patients had significantly higher levels of total bilirubin (57.6 versus 13.1 *μ*mol/L, *P* < .04), AP (216.4 versus 64.0 IU/L; *P* < .02), GGT 273.03 versus 28.96 IU/L; *P* < .004), AST (56.3 versus 24.4; *P* < .05), ALT (95.42 versus 28.9 IU/L; *P* < .009), as well as the higher levels of CRP (28.57 versus 4,84 mg/L; *P* < .003) in comparison with group chosen for laparoscopic cholecystectomy.

In both groups, the patients with acquired or inherited immunodeficiencies and those who developed postoperative complications were excluded from the study. Blood samples were collected preoperatively and on postoperative day 1 and tested for hematological and immunological parameters. Twenty healthy persons, consisting of laboratory personnel and blood volunteer donors (10 females and 10 males), corresponding by age and sexes to the examined patients groups were also studied.

### 2.2. Lymphocyte Preparation

Heparinized venous blood (10 mL) was diluted and separated on Lymphoprep (Nycomed Pharmaas, Oslo, Norway) for 20 minutes on 800 G. Cells accumulating at the interface were washed twice in RPMI 1640 and resuspended at a final concentration of 1 × 10^6^ peripheral blood lymphocytes per sample in fluorescent-activated cell sorting (FACS) buffer. Viability was assessed by trypan blue and was always around 98%.

### 2.3. Monoclonal Antibodies (MoAbs)

For staining of surface markers on human T and NK cells, the following phycoerythrin-(PE)- conjugated MoAb, obtained from Becton Dickinson (Mountain View) were used: anti-CD3, anti-CD4, anti-CD8, anti-CD16, and anti-CD56). For the detection of intracellular perforin a murine antihuman perforin MoAb *δ*G9 (MoAb; IgG2b) was purified from Balb/c ascites [16].

### 2.4. Simultaneous Measurement of Cell Surface (CD Markers) and Intracellular Antigen (Perforin) by Flow Cytometry

The method for the simultaneous detection of perforin (intracellular antigen) and cell surface antigens is described in detail elsewhere [17]. Briefly, peripheral blood mononuclear cells (PBMNLC) were aliquoted (10^6^ per aliquot) and washed in FACS buffer, then fixed in 100 *μ*L PBS containing 4% paraformaldehyde, pH 7.4, for 10 minutes at room temperature. After two washes in FACS buffer, the cells were permeabilized with saponin buffer (0.1% saponin) (Sigma, Poole, Dorset) for 20 min at room temperature. Murine monoclonal antibody reacting with human perforin (P) MoAb-G9 (IgG2b, purified from Balb/c ascites) was added to the suspension of cells in saponin, at final concentration of 8–10 *μ*g per sample and incubated for 30 minutes at room temperature. All further steps, secondary antibody, FITC-conjugated goat-antimouse Ig (Becton Dickinson, San Jose, Calif, USA), and rising procedures were performed in Saponin buffer. Cells surface antigens (CD3, CD4, CD8, CD16, and CD56) were then stained in FACS buffer at a final concentration of monoclonal antibody of 10 *μ*g/mL (Becton Dickinson) and 100 *μ*L were added to the pelleted cells. After 30-minutes incubation at +4°C, cells were washed with FACS buffer and stained with avidin-phycoerythrin (20 *μ*g/mL, Becton Dickinson) for 30 min. An irrelevant isotype-matched murine MoAb was used as a negative control. A minimum of 10^4^ cells was analyzed on FACScan (Becton Dickinson).

### 2.5. Liver Functional Tests

The standard preoperative workup for predicting choledocholithiasis in both group of patients included liver function tests and abdominal ultrasound. Liver transaminases (ALT, AST), liver cholestasis tests (gamma-glutamyl transpeptidase-GGT, and alkaline phosphatase-ALP) and liver clearance tests (total and conjugated bilirubin-BIL) were determined by enzymatic calorimetric methods, using routine laboratory techniques with Roche reagents in a Roche-Hitachi Modular-P800 clinical chemistry module (Roche, Mannhein, Germany).

### 2.6. Statistical Analysis

Statistical significance was calculated by two-tailed Student's *t*-test for paired samples, and by Mann-Whitney *U*-test for the evaluation of differences between the groups. Test of correlation and linear regression analyses were made by statistical software Statistics for Windows, Kernel release 5.5 (StatSoft, Inc., Tulsa, OK). Linear regression equations were generated using a Pearson-Product moment correlation and analyzed for differences of covariance. *P* values of .05 or less were considered statistically significant. Data are reported as mean ± SEM or as median values, with 25th–75th percentiles in parentheses.

## 3. Results

Immune status of patients undergoing classic, open (OC), or laparoscopic (LC) cholecystectomy was evaluated before and 24 hours after the operations. Comparison of the data was then made between the pre- and postoperative values in the same group, between the preoperative values in the OC and LC groups as well as between the preoperative values in groups of patients elected for operations and the control group, consisting of healthy blood donors of corresponding age. Analysis included standard determination of surface antigens on lymphatic cells and two color analysis, with simultaneous determination of surface markers and intracellular content of perforin. 

### 3.1. Peripheral Blood Lymphocyte Subpopulations in Patients Undergoing to Open or Laparoscopic Cholecystectomy

**Figure 2:**
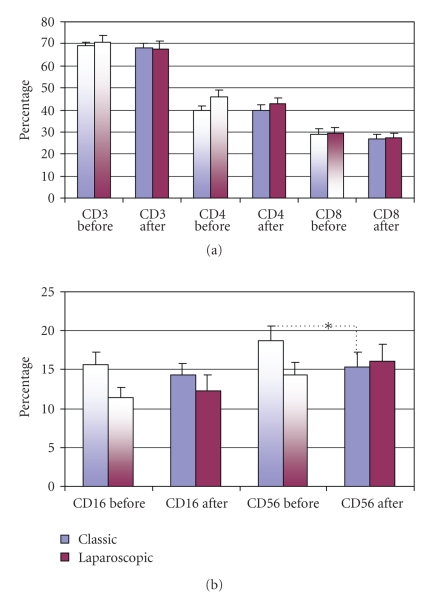
Percentage of surface marker positive T lymphocytes (a) and NK cells (b) (mean percentage ± S.E.M.) in peripheral blood from patients subjected to classic or laparoscopic cholecystectomy before (two-colored columns) and after operation (one-colored column). *Statistical significance between the preoperative and postoperative values *P  < * .*05.

Standard immunological tests revealed that preoperative numbers of T lymphocytes (CD3^+^, CD4^+^, and CD8^+^) and NK cells (CD16^+ ^and CD56^+^) in both groups were in normal ranges for healthy, adult people. Furthermore, no differences were found in preoperative values of both T (Figure 2(a)) and NK cells (Figure 2(b)) in blood of patients elected for OC and LC groups as well as in the postoperative values of all T lymphocytes and CD16^+^ NK cells. Classic cholecystectomy, however, slightly decreased the percentage of CD56^+ ^NK cells (18.7% versus 15.4%; *P* < .05), as shown on Figure 2(b).

### 3.2. Perforin Positive Peripheral Blood Lymphocytes

**Figure 3:**
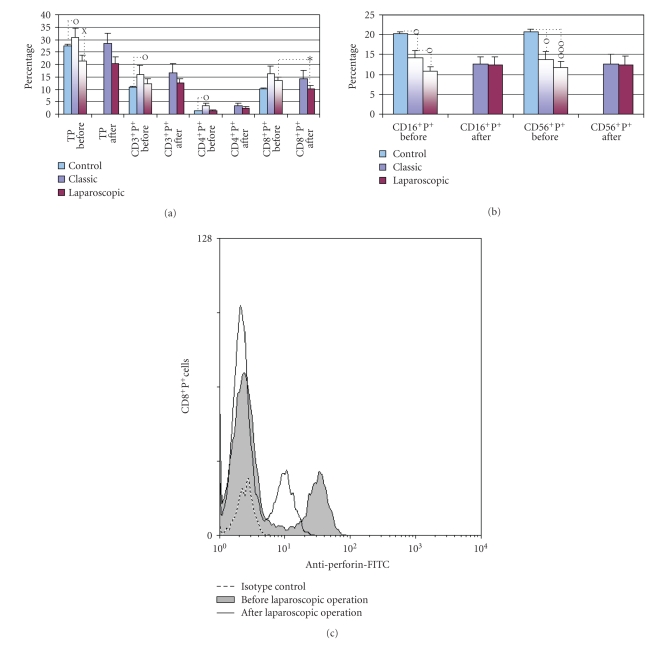
Percentage of total number of perforin positive cells (TPs) and double positive (perforin positive (P^+^) and surface marker positive) T lymphocytes (a) or NK cells (b) (mean percentage ± S.E.M.) in peripheral blood from patients subjected to classic or laparoscopic cholecystectomy before (two-colored columns) and after operation (one-colored column) compared to healthy control (first, blue column). O**-**statistical significance toward healthy control; X-statistical significance between the preoperative values in classic and laparoscopic cholecystectomy; *statistical significance between the preoperative and postoperative values in classic or laparoscopic cholecystectomy. One mark *P * < * .*05, two marks *P * < * .*01, three marks *P* < .001. (c) Representative flow cytometric profile of perforin expression on the gated CD8^+^ PBMNLC before (shadow area) and 24 hours after (solid line) laparoscopic cholecystectomy. Isotype control is shown as dotted line.

Data were then analyzed by double labeling of CD surface marker and perforin and presented as a fraction of total peripheral blood lymphocytes containing perforin (TP^+^ cells) and as a percentage of double positive T (Figure 3(a)) or NK cells (Figure 3(b)). 

The significant postoperative difference was found only after LC, which decreased the fraction of cytotoxic CD8^+^P^+ ^cells from 13.5 to 10.2% (*P* < .05), as it is presented on Figure 3(a) and on representative histogram (Figure 3(c)), showing perforin expression on the gated CD8^+ ^T cells, before and after LC. 

Comparison with healthy control revealed, however, that preoperative values of TP^+^ and CD3^+^P^+^ cells in patients elected for OC were significantly higher than normal values (30.78% and 16.05% versus 27.3% and 10,68%, resp.; *P* < .05) (Figure 3(a)), as well as that all patients elected for operations had, preoperatively, lower fractions of perforin positive NK cells (Figure 3(b)), since in OC group CD16^+^P^+^ cell fraction fell from 20.16% to 14.14% (*P* < .05) and CD56^+^P^+^ cells from 20.74% to 13.63% (*P* < .05) and in LC group to 10.71% (*P* < .05) and to 11.64% (*P* < .001), respectively. 

**Figure 4:**
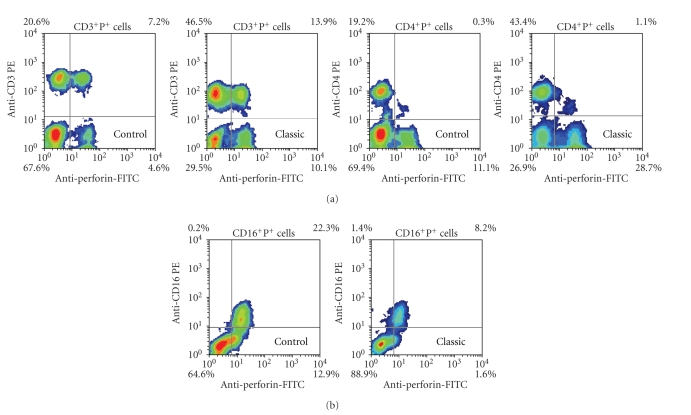
Representative two color analysis of PBMNLC for perforin (horizontal) and cell surface phenotype (vertical) in healthy persons and patients elected for classic cholecystectomy. (a) Subpopulations of T lymphocytes (CD3^+^P^+^ and CD4^+^P^+^); (b) subpopulation of NK cells (CD16^+^P^+^).

Increased expression of perforin in T cells (CD3^+^P^+^ and CD4^+^P^+^) and lower expression of perforin in NK cells (CD16^+^P^+^) in preoperative blood of patients elected for classic cholecystectomy versus control subjects are presented also by the representative density plots (Figure 4(a) and 4(b), resp.).

### 3.3. Level of Perforin Protein Expression in T and NK Cell Subsets

**Figure 5:**
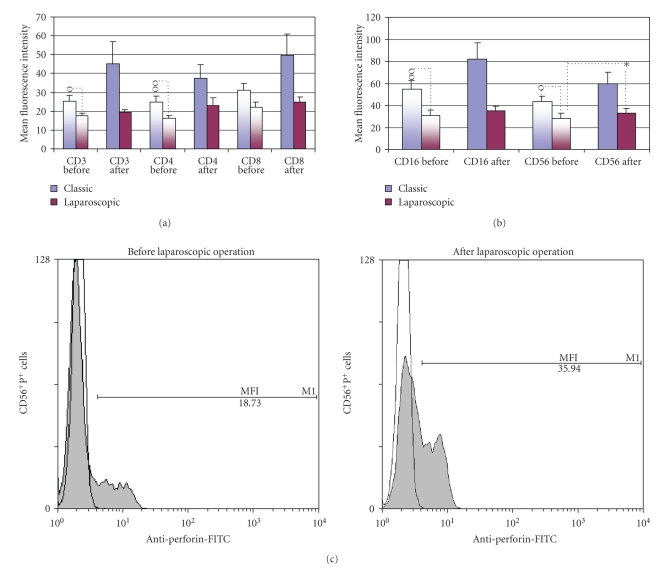
The levels of P expression (mean fluorescence intensity; MFI values) for (a) T lymphocytes (CD3^+^P^+^) and their subpopulations (CD4^+^P^+^ and CD8^+^P^+^), and (b) NK cells (CD16^+^P^+^ and CD56^+^P^+^) in peripheral blood from patients subjected to classic or laparoscopic cholecystectomy before (two-colored columns) and after the operation (one-colored column). O-statistical significance between the preoperative values in classic and laparoscopic cholecystectomy; *statistical significance between the preoperative and postoperative values in classic or laparoscopic cholecystectomy. One mark *P * < * .*05, two marks *P * < * .*01. (c) Representative flow cytometric profile of perforin expression on the gated CD56^+^ PBMNLC before and 24 hours after laparoscopic cholecystectomy. The shadow area was perforin expression while the nonshadow area was isotype control. The amount of perforin per cell is indicated by the mean fluorescence intensity (MFI).

**Figure 6:**
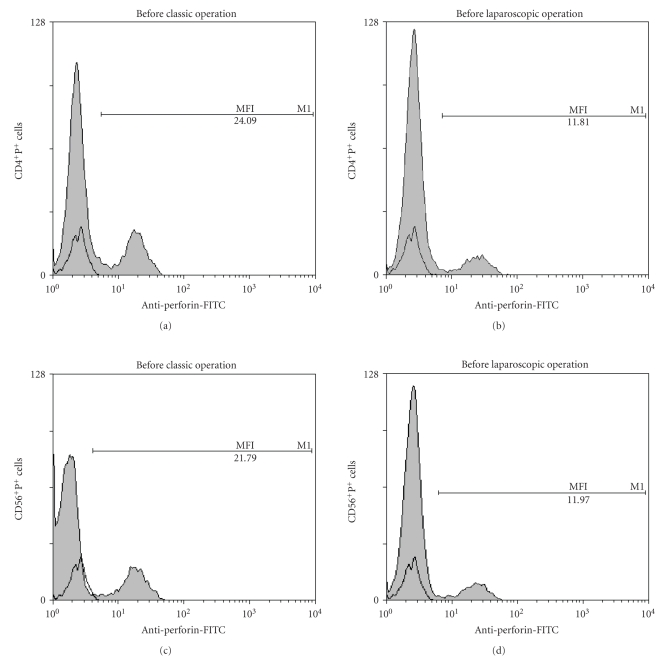
Representative flow cytometric profile of perforin expression on the gated CD4^+^ and CD56^+^ PBMNLC before classic and laparoscopic cholecystectomy. The shadow area was perforin expression while the nonshadow area was isotype control. The amount of perforin per cell is indicated by the mean fluorescence intensity.

The procedure of double labeling of peripheral blood lymphocytes by flow cytometry permitted also the measurement of mean fluorescence intensity (MFI), which correlates with a number of perforin molecules present in the cells [17]. By applying standard parameters for FSC, SSC, FL1 and FL2, we thus, analyzed MFI for perforin in subsets of T lymphocytes (CD3^+^P^+^, CD4^+^P^+^, CD8^+^P^+^) and NK cells (CD16^+^P^+^ and CD56^+^P^+^). As presented on Figure 5(b) and on representative histogram (Figure 5(c)) we noticed slight upregulation of perforin content only after LC in CD56^+^P^+^cells (*P* < .05). The data, however, clearly showed that lymphatic subpopulation (CD3, CD4, CD16 and CD56) of patients undergoing the classic cholecystectomy contained, preoperatively, the higher quantities of perforin in their granules in comparison with patients selected for LC (Figures 5(a), 5(b), and 6). 

### 3.4. Correlation between the Intensity of Preoperative Cholestasis and Presence of Perforin-Expressing Cells

The findings of the marked changes in the proportions and MFI of perforin-expressing cells in preoperative blood suggested that these events might be related with the previously described greater cholestasis and inflammation in patients elected for classic cholecystectomy (Figure 1). To test this possibility the concentrations of CRP, hepatic enzymes and bilirubin in preoperative sera of all patients undergoing to the operations were, individually, in each person correlated with the percentage of perforin positive cells and with MFI for perforin in lymphocytes subpopulations found in preoperative samples of blood. 

**Figure 7:**
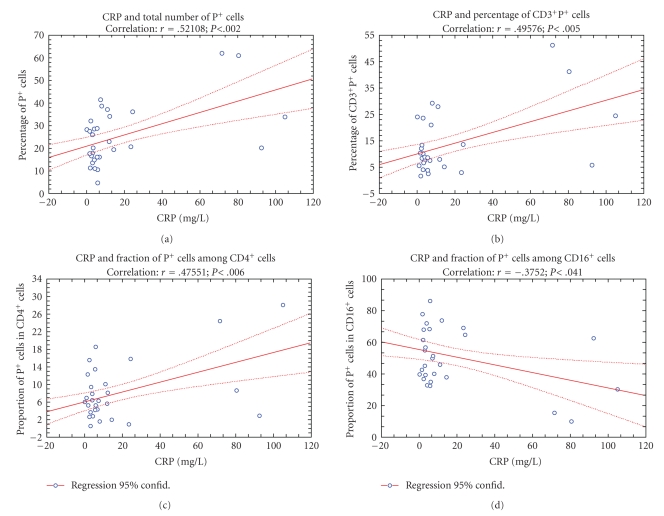
Correlation (*r*) between preoperative serum levels of C-reactive protein (CRP) and total number of perforin positive cells, CD3^+^P^+^ cells and fraction of perforin positive (P+) cells among CD4^+^ T cells, CD8^+^ T cells or CD16^+ ^NK cells in preoperative samples of blood.

It was found (Figure 7) that concentration of CRP was in a very high positive correlation with percentage of total number of perforin positive cells (*r* = 0.5218; *P* < .002), CD3^+^P^+^ (*r* = 0.49576; *P* < .006) and the fraction of P^+^ cells among CD4^+^ subset of cells (*r* = 0.47551; *P* < .006) as well as in the negative correlation with the fraction of P^+ ^cells among CD16^+^ cells (*r* = −0.3752; *P* < .041). 

**Figure 8:**
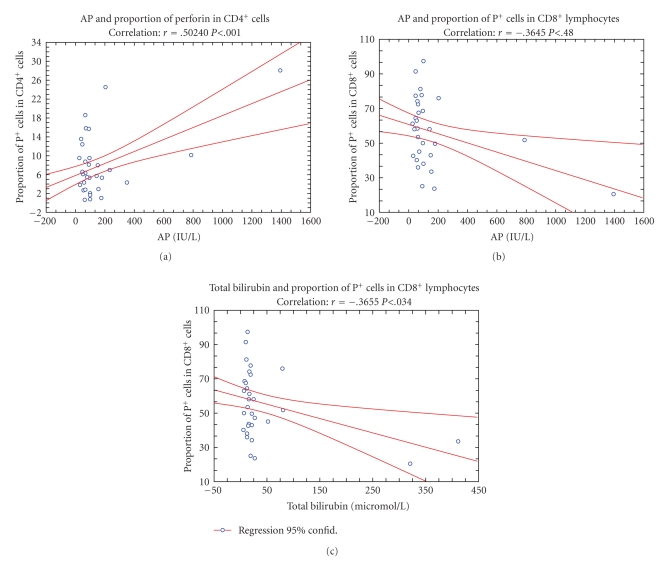
Correlation (*r*) between preoperative serum levels of alkaline phosphatase (AP) and total bilirubin and the proportion of CD4^+^P^+ ^cells or fraction of perforin positive (P+) cells inside the CD8^+^ subpopulation of T cells, respectively.

The data (Figure 8) revealed also the presence of positive correlation between the concentration of AP in serum and proportion of CD4^+^P^+^ cells (*r* = 0.50240; *P* < .001) as well as the presence of negative correlation between AP and proportion of P^+ ^cells among the CD8^+ ^cells (*r* = −0.3645; *P* < .04), and between the concentration of total bilirubin and proportion of P^+^ cells in CD8^+^ cells (*r* = −0.3655; *P* < .034). 

**Figure 9:**
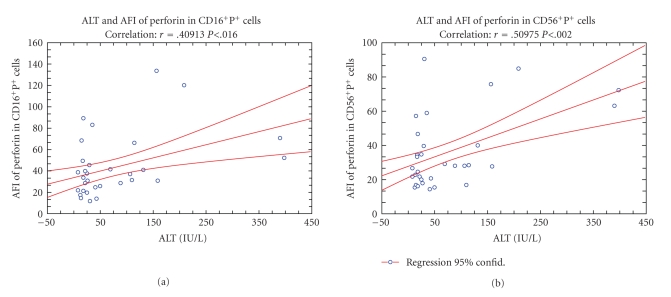
Correlation (*r*) between preoperative serum level of alanine aminotransferase (ALT) and average fluorescence intensity (AFI) of perforin in CD16^+^ and CD56^+^ NK cells.

However, although these data suggested that cholestasis and inflammation have induced a switch in immune response toward the CD4^+^P^+ ^cells, it was also found that higher concentrations of ALT in sera increased the MFI of perforin in CD16^+^ (*r* = 0.409; *P* < .016) and CD56^+ ^cells (*r* = 0.509; *P* < .002), suggesting that remaining NK cells, owing to the increased content of P probably obtained a higher cytotoxic activity (Figure 9).

## 4. Discussion

Trying to contribute to the elucidation of profound immunological dysfunctions induced by surgical trauma [1–11] and mechanisms that regulate the P expression in cytolytic cells [12–15], in this study we analyzed primarily the changes in perforin expressing peripheral blood lymphatic cells after OC and LC. The data showed that in early postoperative period, the type of operation minimally affected the P-related events, since only LC decreased the percentage of circulating CD8^+^P^+ ^cells (Figures 3(a), 3(c)) and increased the MFI of perforin in CD56^+ ^cells (Figures 5(b), 5(c)), suggesting that the pneumoperitoneum with insufflations of CO2 had a stimulating effect on cytolytic activity of NK cells and contributed to distribution of CTL in the tissue. Changes might be related with lower operative stress, extracellular acidification or selective activation of different macrophage subpopulation [1–11] but need further characterization, particularly, owing to our findings that immune outcome of cholecystectomy might be markedly influenced by the intensity of preoperative inflammation and cholestasis. In our study this, obviously, influenced the number of perforin expressing cells in both groups of patients since they, preoperatively, had significantly lower proportions of CD16^+^P^+^ and CD56^+^P^+^ cells (Figures 3(b) and 4(b)). Similarly, augmented proportions of TP^+^ cells, CD3^+^P^+^ cells and CD4^+^P^+^ cells (Figures 3(a) and 4(a)) were found in patients elected for OC, which preoperatively, had higher preoperative levels of CRP, AP, B, AST and ALT (Figure 1), suggesting that preoperative inflammation and cholestasis affected the perforin-related events. The hypothesis was confirmed by linear regression analyses, made in all patients elected for the operations, which clearly showed that the concentration of CRP in the serum was in high positive correlation with the percentage of TP^+^ cells, CD3^+ ^P^+^ and CD4^+^P^+^ cells and in negative correlation with the fraction of P^+^ cells among the CD16^+^ NK cells (Figure 7). Moreover, an additional effect of cholestasis was confirmed by finding of positive correlation between AP in the serum and fraction of CD4^+^P^+^ cells, and negative correlation of both AP and bilirubin with fraction of P^+ ^ cells among CD8^+^ cells (Figure 8). Taken together, the data imply that, in patients with gallstones, the inflammation and chronic cholelithiasis had particularly stimulated the generation of cytotoxic CD4^+^P^+^ cells, decreasing simultaneously the circulating levels of CD8^+^P^+^ T cells and CD16^+^P^+ ^NK cells (Figures 3, 4, 6, and 7), probably owing to their greater homing into the insulted liver tissue. However, based on the fact that ALT was in high positive correlation with MFI of perforin in CD16^+^ and CD56^+ ^cells (Figure 9), we speculated also that hepatic injury might increased the content of perforin per cell in remaining circulating NK cells. 

The data are in high agreement with current knowledge about the influence of inflammation and injuries on cytotoxic cells of innate [18, 19] and adaptive immunity [20, 21] and point to the liver as an important organ for maintenance of self-tolerance and elimination of potentially dangerous cells that arose during the cholestasis and hepatic damages in patients with gallstones. The mechanisms are unclear, since the consequences of obstructive jaundice are complex and involve a number of immune effectors that contribute to a variety of immunological derangements [22, 23] as well as to hepatic regeneration [24, 25] and fibrosis [26]. In this context it could be speculated that destruction of intrahepatic bile ducts or the appearance of aberrant cholangiocytes has initially stimulated the intrahepatic NK cells, leading to cholangiocyte killing and apoptosis by the granzyme/perforin pathway or by activation of death receptors belonging to the tumor necrosis factor (TNF) receptor superfamily and TRAIL, as it was reported in the bile duct-ligated mice [27] and in patients with the primary biliary cirrhosis, where the activation of hepatic NK cells led to exacerbated damage on liver tissues [23, 28, 29]. Noteworthy, higher cytotoxicity might be induced by activation of several classes of killer lectin-like receptors (e.g., NKG2D and natural cytotoxicity receptors), that are commonly expressed by human NK cells as well as by TCR*γδ* ^+^ and TCR*αβ*CD8^+^ T lymphocytes and specifically interact with stress-inducible molecules, such as MICA, MICB, and ULBP, which might be displayed by “stressed” or “altered” self cells and by virus-infected cells [30]. Furthermore, since cholestatic jaundice is often followed by bacteriemia [22, 23], for our data showing the correlation of CD4^+^P^+^ cells with the serum levels of CRP and AP (Figures 7 and 8) might be relevant that in inflammatory bowel diseases with uncontrolled immune responses to bacterial flora were found CD4 (+) NKG2D (+) T-cell clones that were involved in perforin-mediated killing of the targets expressing MICA [31]. 

It should be, however, emphasized that CD4^+^P^+^ cells T cells, activated in the liver, might belong also to CD4^+^NKT cells or to CD4^+^CD25^+^ regulatory T cells, since these autoreactive T cells clones also participate in perforin-mediated killing. The former, which might be activated by glycolipid antigens, are characterized also by rapid activation and secretion of large amounts of numerous types of cytokines that in turn interact with a multitude of other cell types [32–35], while the latter are known to participate in the control of autoantigen recognition and the development of autoreactive T cells clones, by mechanisms that include direct killing of cytotoxic cells through cell-to-cell contact, inhibition of cytokine production by cytotoxic cells and direct secretion of immunomodulatory cytokines, such as TGF-*β* and interleukin-10 [36–39], acting together with other CD4^+^ cells as crucial regulators of adaptive and innate immune responses [40].

In conclusion, our data show that in early postoperative period only laparoscopic cholecystectomy moderately affected perforin-expressing cells in peripheral blood, decreasing the percentage of CD8^+^P^+^ cells and increasing the content of perforin in CD56^+^ cells. However, pointing to the complexity of physiological and immunological derangements induced by cholelitiasis and obstructive jaundice, we also show that preoperative cholestasis and hepatic injury, might have a marked influence on perforin-related aspects of cytotoxic T and NK cells and final interpretation of data obtained by classic or laparoscopic cholecystectomy.

## Figures and Tables

**Table 1:** Demographic and surgery-related data in patient groups for classic and laparoscopic cholecystectomy.

	Classic cholecystectomy	P	Laparoscopic cholecystectomy
Number of patients	23	—	21
Ratio Female/male	13/10	—	13/8
Age-mean (min-max)	61.8 (31–85)	0.23 n.s.	55.5 (28–82)
Duration of anesthesia (min)	90 (74–130)	0.38 n.s.	120 (90–150)
Duration of operation (min)	65 (35–120)	0.45 n.s.	90 (40–150)

## Abbreviations

ALT: Alanine amino-transferase
AP: Alkaline phosphatase
AST: Aspartate amino-transferase
MFI: Mean fluorescence intensity
CRP: C-reactive protein
CTL: Cytotoxic T lymphocytes
BIL: Direct bilirubin
GGT: Gamma-glutamyl transpeptidase
IL-6: Interleukin-6
LC: Laparoscopic cholecystectomy
NK: Natural killer cells
NKT: Natural killer T cells
OC: Open cholecystectomy
P: Perforin
PBMCs: Peripheral blood mononuclear cells
Treg: Regulatory T cells
TNF-alpha: Tumor necrosis factor alpha.
